# The 5-HT7 receptor as a potential target for treating drug and alcohol abuse

**DOI:** 10.3389/fnins.2014.00448

**Published:** 2015-01-13

**Authors:** Sheketha R. Hauser, Peter B. Hedlund, Amanda J. Roberts, Youssef Sari, Richard L. Bell, Eric A. Engleman

**Affiliations:** ^1^Department of Psychiatry, Indiana University School of MedicineIndianapolis, IN, USA; ^2^Department of Molecular and Cellular Neuroscience, The Scripps Research InstituteLa Jolla, CA, USA; ^3^Molecular and Cellular Neuroscience Department, Mouse Behavioral Assessment Core, The Scripps Research InstituteLa Jolla, CA, USA; ^4^Department of Pharmacology, College of Pharmacy and Pharmaceutical Sciences, University of ToledoToledo, OH, USA

**Keywords:** serotonin-7 (5-HT7), alcohol abuse, drug abuse, mesocorticolimbic dopamine system, genetics, pharmacogenetics, selective breeding

## Abstract

Alcohol and drug abuse take a large toll on society and affected individuals. However, very few effective treatments are currently available to treat alcohol and drug addiction. Basic and clinical research has begun to provide some insights into the underlying neurobiological systems involved in the addiction process. Several neurotransmitter pathways have been implicated and distinct reward neurocircuitry have been proposed—including the mesocorticolimbic dopamine (MCL-DA) system and the extended amygdala. The serotonin (5-HT) neurotransmitter system is of particular interest and multiple 5-HT receptors are thought to play significant roles in alcohol and drug self-administration and the development of drug dependence. Among the 5-HT receptors, the 5-HT7 receptor is currently undergoing characterization as a potential target for the treatment of several psychiatric disorders. Although this receptor has received only limited research regarding addictive behaviors, aspects of its neuroanatomical, biochemical, physiological, pharmacological, and behavioral profiles suggest that it could play a key role in the addiction process. For instance, genomic studies in humans have suggested a link between variants in the gene encoding the 5-HT7 receptor and alcoholism. Recent behavioral testing using high-affinity antagonists in mice and preliminary tests with alcohol-preferring rats suggest that this receptor could mediate alcohol consumption and/or reinforcement and play a role in seeking/craving behavior. Interest in the development of new and more selective pharmacological agents for this receptor will aid in examining the 5-HT7 receptor as a novel target for treating addiction.

## Introduction

The neurotransmitter serotonin (5-HT) plays a major a role in a number behavioral and psychophysiological functions such as behavioral inhibition, appetite regulation, mood, cognitive functions, thermoregulation, and addictive behaviors. Relevant to the present review, dysregulation of the 5-HT system has been implicated as a factor in developing alcohol addiction (Engleman et al., 2008; Hayes and Greenshaw, 2011; Kirby et al., 2011; Sari, 2013). For instance, alterations in the 5-HT system are believed to mediate some of alcohol's effects in rat lines selectively bred for high alcohol consumption (c.f., Bell et al., 2012) and alcoholic individuals with a polymorphism of the 5-HT transporter can respond favorably to certain medications (Johnson, 2010; Johnson et al., 2011). Regarding the effects of alcohol, acute exposure increases 5-HT activity/neurotransmission (McBride et al., 1993; Smith and Weiss, 1999) but appears to reduce the firing rate (Pistis et al., 1997) or excitability (Maguire et al., 2014) of 5-HT neurons. It has been suggested that alcohol-induce enhancement of 5-HT release may be due to selective effect on neuronal terminals (Pistis et al., 1997) whereas alcohol-induced decreases of the firing rate (Pistis et al., 1997) and excitability (Maguire et al., 2014) of 5-HT neurons within dorsal raphe nucleus (DRN) may be selective for somatodendritic region of 5-HT neurons (Pistis et al., 1997). Chronic exposure of alcohol results in the development of tolerance to 5-HT neurotransmission (Smith and Weiss, 1999). In addition, outbred Wistar rats show rapid tolerance to elevations in mesolimbic extracellular 5-HT levels induced with systemic ethanol administration (Bare et al., 1998), whereas alcohol-preferring rats (P-rats) selected for alcohol preference do not (Thielen et al., 2002). Moreover, alcohol-nonpreferring (NP) rats selected for alcohol non-preference show no 5-HT response to ethanol in the same dose range (Thielen et al., 2002). Clinical and/or pre-clinical studies have reported deficiencies of 5-HT and/or its major metabolite 5-HIAA in the brains of human alcoholics (Schmidt et al., 1997; Pivac et al., 2004) and P-rats (Murphy et al., 1987; Zhou et al., 1991; McBride et al., 1993) as well as other rats selectively bred for an alcohol preference over water (c.f., Bell et al., 2012). Pharmacologically, treatments that reduce 5-HT neurotransmission can elevate self-administration of alcohol (Lyness and Smith, 1992; Ciccocioppo et al., 1999), while treatment with antidepressants that increase 5-HT central nervous system (CNS) levels reduce craving and withdrawal-associated behaviors (c.f., Goodman, 2008). Therefore, it has been proposed that modulation of the 5-HT system is a viable therapy for alcoholism in a sub-set of patients, suggesting its role in pharmacogenetics (Johnson, 2004, 2010; Wrase et al., 2006).

## The 5-HT7R: molecular structure, system transduction, distribution and pharmacological actions in the CNS

There are seven families of 5-HT receptors (5-HT1–7) and at least 14 distinct 5-HT receptor subtypes (Barnes and Sharp, 1999), which makes the task of understanding the extent to which each of the 5-HT receptor subtypes mediate addictive behaviors a complex one. The most recently discovered 5-HT receptor is the 5-HT7 receptor which was identified in 1993 (Bard et al., 1993; Lovenberg et al., 1993; Ruat et al., 1993). The 5-HT7 receptor (5-HT7R) has been cloned for the human (Bard et al., 1993), rat (Lovenberg et al., 1993; Meyerhof et al., 1993; Ruat et al., 1993), mouse (Plassat et al., 1993), guinea pig (Tsou et al., 1994), and frog (Nelson et al., 1995). The 5-HT7R is a polypeptide of 448 amino acids in the rat (Ruat et al., 1993). Other studies have demonstrated that the receptor is constituted of either 404 (Shen et al., 1993) or 435 amino acids in rats (Lovenberg et al., 1993). It has been suggested that these differences might be due to the presence of an intron in the region coding for the secondary putative boucle (Shen et al., 1993), or the presence of a secondary intron on the C-terminus of the protein (Ruat et al., 1993). In addition, studies have revealed the existence of four isoforms of the 5-HT7R in humans and rats, which are produced through alternative splicing (Heidmann et al., 1997). Also, the 5-HT7R has a long C-terminal portion making its homolog sequence with other cloned receptors limited (<40%) (Meyerhof et al., 1993; Plassat et al., 1993; Ruat et al., 1993; Shen et al., 1993).

The 5-HT7R is a G-protein-coupled receptor (GPCR) with positive coupling to adenylate cyclase stimulating the production of cAMP (Bard et al., 1993; Lovenberg et al., 1993; Ruat et al., 1993). Parallel research indicated that its activation, in COS-7 or HEK-293 transfected cell lines, induced increases in adenylate cyclase activity (Lovenberg et al., 1993; Plassat et al., 1993; Ruat et al., 1993; Shen et al., 1993). Using RT-PCR analyses, it has been shown that *5htr7* mRNA is expressed in the forebrain, brainstem and cerebellum, as well as in the periphery such as the heart and intestine (Plassat et al., 1993). Northern blot analyses have demonstrated that *5htr7* mRNA is highly expressed in the hypothalamus, thalamus, hippocampus, and brainstem; however, low densities were also found in the cerebral cortex, striatum, and olfactory tubercle of the guinea pig (Lovenberg et al., 1993; Meyerhof et al., 1993; Plassat et al., 1993; Ruat et al., 1993; Shen et al., 1993). Furthermore, ligand binding studies using [3H]-5-carboxamidotryptamine (5-CT) demonstrated that the receptor is localized in cortical layers I–III, septum, thalamus, hypothalamus, hippocampus, amygdala, periaqueductal gray matter, and superior colliculus of the rat (Gustafson et al., 1996).

In general, 5-HT7Rs are highly expressed in specific brain areas where they are believed to mediate certain behavioral and physiological functions. 5-HT7Rs are expressed in the thalamus (sleep, epilepsy), hypothalamus (circadian rhythm, thermoregulation, stress), hippocampus (memory, learning), amygdala (emotional processes, motivation), and cortex (mood, cognition, sleep) in humans and rodents (To et al., 1995; Thomas et al., 2002; Martín-Cora and Pazos, 2004; Varnas et al., 2004; Horisawa et al., 2013). Autoradiography techniques using [3H]SB-269970 to selectively label 5-HT7Rs, also found 5-HT7Rs in brainstem nuclei including the ventral tegmental area (VTA: reward, addiction), the dorsal raphe nucleus [DRN: circadian rhythm (along with the suprachiasmatic nucleus: Lovenberg et al., 1993; Prosser et al., 1993; Ying and Rusak, 1997; Horikawa et al., 2000; Ehlen et al., 2001; Yu et al., 2001; Antle et al., 2003; Sprouse et al., 2004) as well as mood], and the substantia nigra (movement, mood) in humans (Varnas et al., 2004). A recent autoradiography study with improved sensitivity in detection of [3H]SB-269970 showed that 5-HT7Rs are expressed in the nucleus accumbens (ACB: reward, addiction), substantia nigra and caudate putamen (movement) as well (Horisawa et al., 2013). 5-HT7Rs are localized on gamma-aminobutyric acid (GABA) interneurons or on glutamate terminals within the CNS (Lovenberg et al., 1993; Harsing et al., 2004; Hedlund, 2009). Regarding general 5-HT7 receptor function, researchers have found that 5-HT7 receptors are involved in thermoregulation (Hagan et al., 2000; Thomas et al., 2003; Hedlund and Sutcliffe, 2004; Matthys et al., 2011), circadian rhythmicity (Matthys et al., 2011), cognitive functions (i.e., learning, memory, attention: Yau et al., 2001; Hedlund and Sutcliffe, 2004; Meneses, 2004), and psychiatric disorders (i.e., anxiety, depression and psychosis: Guscott et al., 2005; Hedlund et al., 2005; Wesolowska et al., 2006a,b; Mnie-Filali et al., 2011).

Since the discovery and successful cloning of 5-HT7 receptors, several 5-HT7R antagonists and agonists have been developed. The 5-HT7R has strong affinity for [^3^H]5-HT, [^125^I]LSD and 5-CT (Lovenberg et al., 1993; Meyerhof et al., 1993; Plassat et al., 1993; Ruat et al., 1993; Shen et al., 1993). These studies also demonstrated that the receptor has strong affinity for neuroleptics, such as (+)butaclamol and clozapine, and antidepressants suggesting a role in certain psychiatric disorders (Plassat et al., 1993; Roth et al., 1994; Mullins et al., 1999). The quest for selective 5-HT7R antagonists (Table 1) has led to the development of LY215840 (Cushing et al., 1996), SB-258719 (Forbes et al., 1998), DR4004 (Kikuchi et al., 1999), SB-269970 (Lovell et al., 2000), and SB-656104-A (Forbes et al., 2002). One of the most useful 5-HT7R antagonists discovered to date is SB269970 which has been widely used to map 5-HT7R distribution in the brain as well as studying its functional and behavioral effects. Regarding 5-HT7R agonists, 8-OH-DPAT, which was initially considered a 5-HT1A agonist, was later discovered to also be an effective 5-HT7R agonist (Dompert et al., 1985; Ruat et al., 1993; Shen et al., 1993; Hedlund and Sutcliffe, 2004). Subsequently, the need for more selective agonists (Table 1) led to the development of AS-19 (Brenchat et al., 2009), MSD-5a, (a partial agonist) (Brenchat et al., 2009), LP-44 (Leopoldo et al., 2004), LP-12 (Leopoldo et al., 2007), LP-211 (Leopoldo et al., 2008; Hedlund et al., 2010), E-55888 (Brenchat et al., 2009), and E-57431(Brenchat et al., 2010).

**Table 1:** **Representation of 5-HT7 antagonist and agonist that are currently being used in research**.

**Drug**	**Pharmacology**	**Receptor affinity**	**References**
LY215840	Antagonist	5-HTI (*K_i_* = 14.7 nm) 5-HT2A (*K_i_* = 19.6 nm) 5-HT2B (*K_i_* = 1.89 nm) 5-HT2C (*K_i_* = 4.26 nm)	Cushing et al., 1996
S0-258719	Antagonist	5-HT7 (*K_i_* = 31.6 nM)	Forbes et al., 1998
DR4004	Antagonist	5-HT7 (p*K_i_* = 8.67) 5-HT2A (p*K_i_* = 6.84)	Kikuchi et al., 1999
SB-269970	Antagonist	5-HT7 (p*K_i_* = 8.9) 5-HTI A (p*K_i_* < 5)	Lovell et al., 2000
SB-656104-A	Antagonist	5-HT7 (p*K_i_* = 8.7) αlb (p*K_i_* < 5)	Forbes et al., 2002
8-0H-DPAT	Agonist	5-HTIA (*K_i_* = 0.4 nM) 5-HT7 (*K_i_* = 35–52 nM)	Dompert et al., 1985; Shen et al., 1993; Ruat et al., 1993; Hedlund and Sutcliffe, 2004
MSD-5a	Partial agonist	5-HT7 (*K_i_* = 0.6 nM) 5-HTIA(*K_i_* = 16 nM) 5-HT2A (*K_i_* = 320 nM)	Brenchat et al., 2009
LP-44	Agonist	5-HT7 (*K_i_* = 0.22 nM) 5-HTIA (*K_i_* = 52.7 nM)	Leopoldo et al., 2004
LP-12	Agonist	5-HT7 (*K_i_* = 0.13 nM) 5-HTI A (*K_i_* = 60.9 nM)	Leopoldo et al., 2007
I.P-211	Agonist	5-HT7 (*K_i_* = 0.58 nM) 5-IITIA (*K_i_* = 188 nM)	Leopoldo et al., 2008; Hedlund et al., 2010
AS-19	Agonist	5-HT7 (*K_i_* = 0.6 nM) 5-HTI A (*K_i_* = 89.7 nM)	Brenchat et al., 2009
E-55888	Agonist	5-HT7 (*K_i_* = 2.5 nM) 5-HTIA (*K_i_* = 700 nM)	Brenchat et al., 2009
E-57431	Agonist	5-HT7 (*K_i_* = 0.47 nM), 5-HTID (*K_i_* = 53 nM) 5-HT2A (*K_i_* = 560 nM)	Brenchat et al., 2010

## Dopamine and 5-HT7

**Figure 1:**
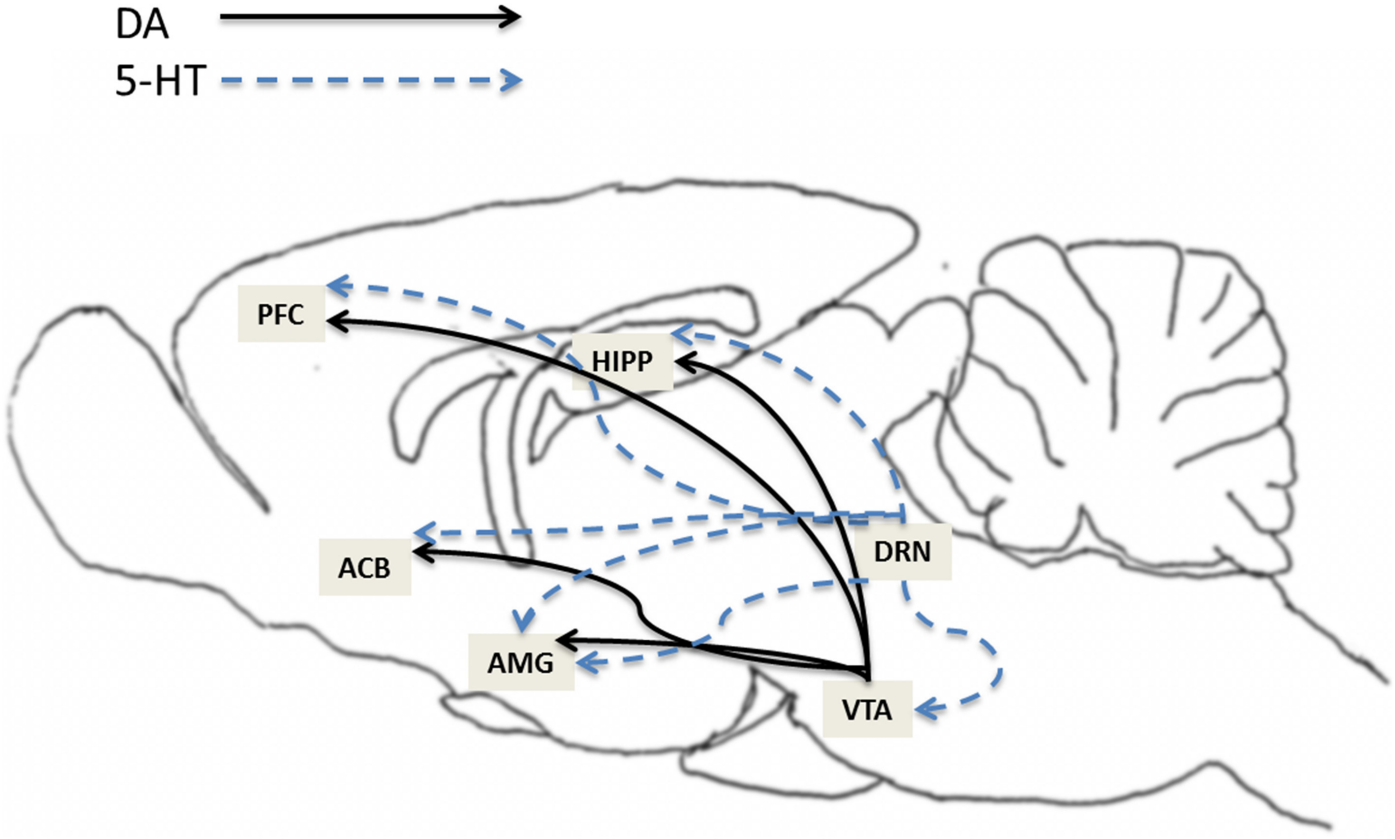
**This illustration depicts the expression of 5-HT7 receptors in brain structures relevant to addiction**. The expression of 5-HT7 receptors has been found within several areas in the mesocorticolimbic dopamine (MCL-DA) reward pathway such as ventral tegmental area (VTA), nucleus accumbens (ACB), amygdala (AMG), hippocampus (HIPP), and prefrontal cortex (PFC). The solids lines represent DA projections from the VTA to the ACB, AMG, HIPP, and PFC. The dotted lines represent 5-HT projections from dorsal raphe nucleus (DRN) to VTA, ACB, AMG, HIPP, and PFC.

The mesocorticolimbic dopamine (MCL-DA) system plays a major role in reward (natural and drugs of abuse), memory, learning, motivation and movement. Numerous studies have reported that activation of the MCL-DA system mediates, at least in part, alcohol and drug addiction. The MCL system consists of DA neurons that originate in the VTA and project to the ACB, amgydala, hippocampus and prefrontal cortex (PFC) (Figure 1). The raphe nucleus, where 5-HT neurons originate, sends 5-HT projections to numerous regions including the VTA, ACB, amygdala, hippocampus and PFC (Figure 1). Moreover, studies have shown that the 5-HT system regulates DA neuronal activity in these subregions of the MCL system (Azmitia and Segal, 1978; Parent et al., 1981; Halliday and Törk, 1987; Herve et al., 1987; Van Bockstaele et al., 1994). For example, 5-HT activates VTA-DA neurons (Pessia et al., 1994), induces DA release in VTA slices (Beart and McDonald, 1982), enhances DA release in the ACB when locally applied to the VTA (Guan and McBride, 1989), potentiates the excitatory actions of alcohol on VTA-DA neurons (Brodie et al., 1995), and increases extracellular DA release in the PFC (Iyer and Bradberry, 1996). In addition, there is evidence that activation of the dorsal raphe nucleus (DRN) can increase extracellular levels of DA in the ACB (Yoshimoto and McBride, 1992).

There are only a few studies that have investigated 5-HT7 receptor involvement in DAergic activity. Proliferating neurospheres of mesencephalic precursors are used to observe the development of cells. SB269970 can increase the generation of DA neurons in proliferating neurospheres of mesencephalic precursors, which can be inhibited by cytosine-D-arabinofuranoside (Ara-C: a cell cycle inhibiter) (Parga et al., 2007). In contrast, a 5-HT7R agonist was shown to block increases in the generation of DA neurons. Interestingly, double labeling for 5-HT7Rs and tyrosine hydroxylase (DA marker) showed that 5-HT7Rs do not appear to be located on DA neurons, whereas double immunolabeling for 5-HT7Rs and glial fibrillary acidic protein (GFAP a marker of astrocytes) or tryptophan hydroxylase (5-HT marker) showed that 5-HT7Rs are located on glia and serotonergic cells (Parga et al., 2007). Taken together, these results suggest that 5-HT7Rs are involved in regulating DA neuronal development following elimination of 5-HT neurons or a reduction of 5-HT levels (Parga et al., 2007). 5-HT7Rs are also involved in DA neuronal firing activity and DA release. An electrophysiological study demonstrated that antagonism of 5-HT7Rs with SB269970 prevented amphetamine-induced inhibition of DA neuronal firing in the VTA (Mnie-Filali et al., 2007). However, administration of a 5-HT7R antagonist alone did not alter the spontaneous activity of DA neurons (Mnie-Filali et al., 2007). DR4004, a 5-HT7R antagonist, can decrease DA and/or 5-HT turnover in the amygdala, suggesting that 5-HT7 receptors may be located presynaptically at DA and 5-HT nerve terminals in the amygdala (Takeda et al., 2005). In other work, Wesolowska and Kowalska (2008) reported that SB-269970 increased DA, norepinephrine (NE) and 5-HT efflux in the PFC (Matthys et al., 2011). It has been suggested that the inhibition of 5-HT7 heteroreceptors on DA and NE neurons may regulate DA and NE release in the PFC, however there is no evidence of this co-localization of 5-HT7Rs (Matthys et al., 2011). Collectively, these studies suggest that activation of 5-HT7Rs may be involved in early neuronal development of the DAergic system and may regulate DA release within the MCL-DA system.

## Gamma-aminobutyric acid (GABA) and 5-HT7 receptors

*Gamma*-aminobutyric acid (GABA) is the principal inhibitory neurotransmitter in the CNS. Research indicates that GABA is involved in alcohol self-administration, the development of tolerance to alcohol's effects, the genetic risk for developing alcohol dependence (AD) and the expression of withdrawal-associated behaviors (Dick and Bierut, 2006; Korpi and Sinkkonen, 2006; Krystal et al., 2006; Kohnke, 2008; Tabakoff et al., 2009; McBride et al., 2010; Enoch et al., 2012; Herman et al., 2013). Although there are currently no published studies that have examined whether 5-HT7Rs mediate these GABAergic effects, it has been reported that 5HT7Rs are found in the GABAergic system. The dorsal raphe nucleus (DRN) sends serotonergic projections to the VTA and ACB and is thought to contribute to addiction behaviors (Tork, 1990; McBride et al., 1990). It has been suggested that 5-HT7Rs are located on GABA neurons (Duncan et al., 2001; Glass et al., 2003; Roberts et al., 2004) within the DRN, but not on the serotonergic neurons themselves (Duncan et al., 2001). Using *in vitro* fast cyclic voltammetry, it appears that GABAergic neurons inhibit neuronal serotonergic activity and 5-HT release in the DRN (Roberts et al., 2004; Matthys et al., 2011). Bicuculline, a GABA_A_ antagonist, inhibited SB-269970 suppression of electrically stimulated 5-HT release in the DRN, suggesting that 5-HT7R activation may inhibit GABA interneurons, leading to a decrease in GABA release with a subsequent reduction in inhibitory tone on 5-HT neurons (Roberts et al., 2004; Matthys et al., 2011). The activation of 5-HT7Rs can enhance GABAergic transmission in the hippocampus (Tokarski et al., 2011) and GABAergic neuronal excitability in the globus pallidus (Chen et al., 2008), whereas 5-HT7Rs in the suprachiasmatic nucleus (SCN) decrease local GABA-dependent excitability (Kawahara et al., 1994). These findings suggest that 5-HT7R modulation of the GABAergic system may vary depending on the neural substrate examined.

## Glutamate and 5-HT7 receptors

Glutamate is the principal excitatory neurotransmitter in the CNS. Alcohol-induced neuroadaptations in glutamate release and receptor up-regulation (Fadda and Rossetti, 1998) are considered to be important factors in the development of tolerance to alcohol's effects, dependence and withdrawal-associated behaviors (Chandler et al., 1998). Research indicates that 5-HT7Rs regulate the glutamatergic system. The activation of 5-HT7Rs increases the firing of glutamatergic neurons in the medial prefrontal cortex (Fan et al., 2011; Pehrson and Sanchez, 2014) and hippocampus (Tokarski et al., 2003; Pehrson and Sanchez, 2014). SB269970 administration significantly reduces MK-801-induced glutamate release in the PFC (Bonaventure et al., 2011). Antagonism of 5-HT7Rs can also reverse 5-HT agonist-induced suppression of glutamate release in the DRN and median raphe nucleus (MRN) (Harsing, 2006; Pehrson and Sanchez, 2014). It has been suggested that there may be 5-HT7 heteroreceptors involved in the inhibition of glutamate release in glutamatergic cortico-raphe projections (Harsing, 2006; Duncan and Congleton, 2010), whereas 5-HT7R enhancement of 5-HT release may be due to GABA-glutamatergic-serotonergic interactions in the DRN (Harsing et al., 2004; Roberts et al., 2004; Tokarski et al., 2012). More recently, Tokarski et al. (2011) have suggested that 5-HT7R activation may enhance GABAergic transmission in the hippocampus via presynaptic 5-HT7Rs on excitatory glutamatergic input to GABAergic interneurons or activation of post-synaptic 5-HT7Rs on the GABAergic interneurons themselves. Given evidence that 5-HT7Rs can modulate glutamatergic output and the putative role glutamatergic systems play in alcohol and drug abuse, manipulation of glutamate neurotransmission with 5-HT7R-associated agents may provide an additional mechanistic approach to develop therapeutic agents targeting addiction.

## A role for 5-HT7 receptors in alcohol and drug abuse

Personality characteristics such as sensation seeking and impulsivity are linked to an increased risk for drug addiction behaviors. Sensation-seeking is defined as voluntary participation in varied, novel, and intense activities without regard to personal risk and it is associated with a greater tendency to abuse drugs and alcohol (Matthys et al., 2011). Ballaz et al. (2007a) investigated the gene expression of *5htr7* mRNA in an animal model in which rats were classified as high responders (HR) that express high levels of novelty seeking and drug-taking behaviors, or low responders (LR) which express the opposite phenotype (Zuckerman and Neeb, 1979; Kabbaj, 2006). The HR rats had significantly lower *5htr7* gene expression in the dorsal hippocampus, intralaminar nucleus, and paraventricular thalamic nucleus than the LR rats (Ballaz et al., 2007a). These results suggest that the low expression of *5htr7* mRNA in HR rats may be involved in increased novelty seeking behavior (Hedlund, 2009). In a subsequent study, novel object discrimination (NOD) task was used to examine attention and memory in HR and LR rats (Ballaz et al., 2007b). LR showed increased exploration of new objects compared to old objects, whereas HR spent the same amount of time exploring new vs. old objects (Ballaz et al., 2007b). Interestingly, prolonged exposure to alcohol has been shown to impair cognitive functions such as attention and memory, and to produce perseveration which is the tendency to continue an activity after the cessation of the original stimulus (Ridley, 1994). Systemic administration of SB269970, a 5-HT7R antagonist, decreased LR exploration of novel objects but did not alter the behavior of HR rats, suggesting that activation of 5-HT7Rs may play an important role in cognitive behaviors such as attention and memory. A knockout mouse study showed that mice without 5-HT7Rs had similar performance in the novel object recognition as that of 5-HT7+/+ mice, but they did exhibit reduced novel location (spatial) recognition (Hedlund, 2009; Sarkisyan and Hedlund, 2009). These authors also reported that administration of SB269970, a 5-HT7R antagonist, also reduced location novelty recognition compared with vehicle-treated mice. Overall, the genetic and pharmacological manipulation of 5-HT7Rs provide further evidence that these receptors may be involved in specific cognitive processes such as attention and location-related memory.

Impulsivity is associated with a loss of behavioral control, which is a prominent trait of attention deficit hyperactivity disorder (ADHD) and can be readily observed in rat models of ADHD (Russell et al., 2000). The transition from moderate alcohol consumption to excessive alcohol consumption has been hypothesized to be based upon a “loss of control,” with reports suggesting that the development and course of alcohol use and dependence is complicated by heightened impulsivity (Miller, 1991; Dom et al., 2006a,b). In particular, impulsivity may be involved in dysregulated alcohol-seeking behavior, relapse and the maintenance of voluntary abstinence (Noel et al., 2007). Leo et al. (2009) investigated the involvement of 5-HT7Rs in an animal model of impulsivity that used a delayed reward task. Results from their study (Leo et al., 2009) led them to hypothesize that 5-HT7Rs play an important role in reward-devaluation processes. These authors also found that administration of the drug methylphenidate (MPD) to rats during adolescence reduced “impulsive” behaviors in adulthood (Leo et al., 2009). Moreover, MPD can increase *5htr7* mRNA expression in the PFC and ACB (Shell and Core), which are key structures in the MCL reward pathway (Leo et al., 2009). In addition, activation of 5-HT7Rs significantly increased neurite length in striatal neuron primary cultures thus suggesting a role for 5-HT7Rs in neuroplasticity (Leo et al., 2009; Matthys et al., 2011). Adriani et al. (2006) also showed that MPD induced an upregulation of *5htr7* mRNA expression in the striatum (c.f., Hedlund, 2009). Therefore, it is possible that 5-HT7R activity could suppress impulsive behaviors by promoting neuronal differentiation in the striatum or other brain regions mediating this behavior (Matthys et al., 2011). Pharmacological data showed that SB-269970 counteracted the effects of MPD leading to an increase in impulsive behaviors, whereas 8-OH-DPAT, a 5-HT7R agonist, reduced impulsive behavior in naïve adolescent and adult rats (Leo et al., 2009), again suggesting that 5-HT7Rs are involved in behavioral self-regulation.

A follow up study using pharmacologic magnetic resonance imaging (phMRI), with SB2690970 and 8-OH-DPAT, found that SB269970 produced a direct and highly selective 5-HT7R blockade in a specific neurocircuit composed of orbital prefrontal cortex (oPFC)-to-ACB projections; whereas 8-OH-DPAT generated a wide spread effect from the dorsal striatum to the medial prefrontal cortex (mPFC) (Canese et al., 2011). These findings provided further evidence that 5-HT7Rs are located within subregions of the MCL reward pathway. In addition, it suggests that two distinct serotonergic sub-pathways may be involved in 5-HT7R activity within the MCL. Collectively, these findings indicate that 5-HT7R activity mediates, at least in part, behavioral self-control further implicating the 5-HT7R as a novel target to reduce maladaptive behaviors associated with alcohol and drug addiction.

There have been a limited number of studies investigating a possible association between 5-HT7Rs and alcohol addiction. Human genetic studies have implicated a *5htr7* polymorphism in a genetic predisposition to develop alcohol dependence (Zlojutro et al., 2011: Kim et al., 2014). Event-related brain oscillations (EROs) are considered to be highly heritable neurophysiological correlates of human perception and cognitive performance with marked deficits displayed in various psychiatric disorders (Zlojutro et al., 2011). ERO deficits have been found among alcohol-dependent and individuals at high-risk to develop this disorder, and these deficits are thought to precede the development of alcoholism Rangaswamy and Porjesz, 2008; Zlojutro et al., 2011. Thus, these authors propose that ERO deficits may serve as an effective endophenotype for alcohol dependence (Zlojutro et al., 2011). Zlojutro et al. (2011) found a *5htr7* polymorphism (the gene is located on chromosome 10q23) that was associated with altered EROs, suggesting that serotonergic activity is involved in the neurophysiological underpinnings of theta EROs. In addition, their findings indicated that this particular *5htr7* polymorphism was associated with (a) an alcohol dependence diagnosis (DSM IV) among case-controls as well as (b) theta ERO reductions among homozygotes for alcohol dependence in both case-control and family-based samples.

In another study, Kim et al. (2014) wanted to replicate the findings of Zlojutro et al. (2011). Their results were consistent with Zlojutro et al. (2011) and indicated that a *5htr7* polymorphism is also associated with the Alcohol Use Disorders Identification Test (AUDIT) which is considered to be a reliable and widely used screening scale for the early detection of alcohol consumption, alcohol dependence, and problems related to drinking (Saunders et al., 1993). Several *5htr7* haplotypes were found to have strong associations with alcohol dependence based on the AUDIT (Kim et al., 2014). In addition, an extensive review of previous findings of genome-wide association studies (GWASs) of alcohol dependence as well as meta-analyses, cis-acting expression of quantitative locus (cis-eQTL) analyses, rat brain transcriptome analyses, bioinformatics analyses and SNP disease association analyses provided further evidence that *5htr7* polymorphisms are likely involved in the risk of alcohol dependence (Zuo et al., 2014).

There is some preclinical evidence that exposure to alcohol vapors can enhance 5-HT7R expression in the brain. Yoshimoto et al. (2012) demonstrated that a single day of alcohol vapor-exposure significantly increased *5htr7* mRNA in the lateral hypothalamus of C57BL/6J mice, while 20 days of exposure enhanced *5htr7* mRNA expression in the ACB and caudate putamen as well. However, antagonism of 5-HT7Rs with SB269970 did not alter alcohol drinking behavior in the animals exposed to alcohol vapor (Yoshimoto et al., 2012). Although the SB269970 did not block alcohol drinking in this particular animal model, it does not rule out 5-HT7R involvement in alcohol addiction behaviors. Utilizing different animal models, different 5-HT7R antagonists (as well as agonists), and/or examining other behaviors such as alcohol-seeking, relapse or reinforcement may reveal a role for 5-HT7Rs in a predisposition for, and/or the development of, alcohol addiction.

## Conclusion

The 5-HT system plays an important role in behaviors associated with addiction processes. Accumulating evidence from multiple disciplines suggests that the 5-HT7R may be involved in various aspects of drug and alcohol consumption as well as reward and reinforcement. The location and neurochemical properties of 5HT7Rs implicate an important role for this receptor in alcohol and drug abuse/dependence. Additional studies are needed to determine the potential that the 5-HT7R holds as a possible molecular target for the treatment of alcohol and drug addiction.

### Conflict of interest statement

The authors declare that the research was conducted in the absence of any commercial or financial relationships that could be construed as a potential conflict of interest.
